# Interferometric Near‐Field Scanning Optical Microscopy via Phase Conjugation of a Multimode Fiber Probe

**DOI:** 10.1002/nap2.70333

**Published:** 2026-10-16

**Authors:** Juntaek Oh, Young‐Ho Jin, Sungsam Kang, Myung‐Ki Kim, Wonshik Choi

**Affiliations:** ^1^ Department of Physics Korea University Seoul Korea; ^2^ KU‐KIST Graduate School of Converging Science and Technology Korea University Seoul Korea

**Keywords:** multimode fiber probe, near‐field phase imaging, phase‐conjugation interferometric NSOM, surface plasmon polaritons, wavefront shaping

## Abstract

Interferometric near‐field scanning optical microscopy (NSOM) enables simultaneous measurement of both the amplitude and phase of the optical near field. In its aperture‐type implementation, the local field is collected through a sub‐wavelength aperture and an optical fiber. The choice of this fiber imposes an inherent trade‐off between signal strength and phase fidelity. Single‐mode fiber probes maintain phase fidelity but suffer from low signal intensity, whereas multimode fiber probes offer higher throughput but introduce severe phase distortions. Here, we present phase‐conjugation interferometric NSOM (PCI‐NSOM), which overcomes this limitation. The output field of the multimode fiber is measured by off‐axis holography, and its phase conjugate is displayed on a spatial light modulator, converting the scrambled speckle into a planar wavefront that focuses to a single point in Fourier space. At the focal pixel of the detector, the correction enhances the intensity by up to ∼165‐fold. We demonstrate PCI‐NSOM by imaging surface plasmon polaritons on a gold grating, obtaining accurate phase maps where uncorrected multimode collection fails through modal dispersion and single‐mode collection through a low signal‐to‐noise ratio. PCI‐NSOM thus provides a practical route to high‐sensitivity phase‐resolved nanoscale optical characterization.

## Graphical abstract

A multimode fiber probe collects far more of the near field than a single‐mode one, but scrambles the phase into speckle. Phase conjugation on a spatial light modulator restores the wavefront, concentrating the signal up to 165‐fold at a single detector pixel and recovering the full phase of propagating surface plasmon polaritons where conventional near‐field detection fails.

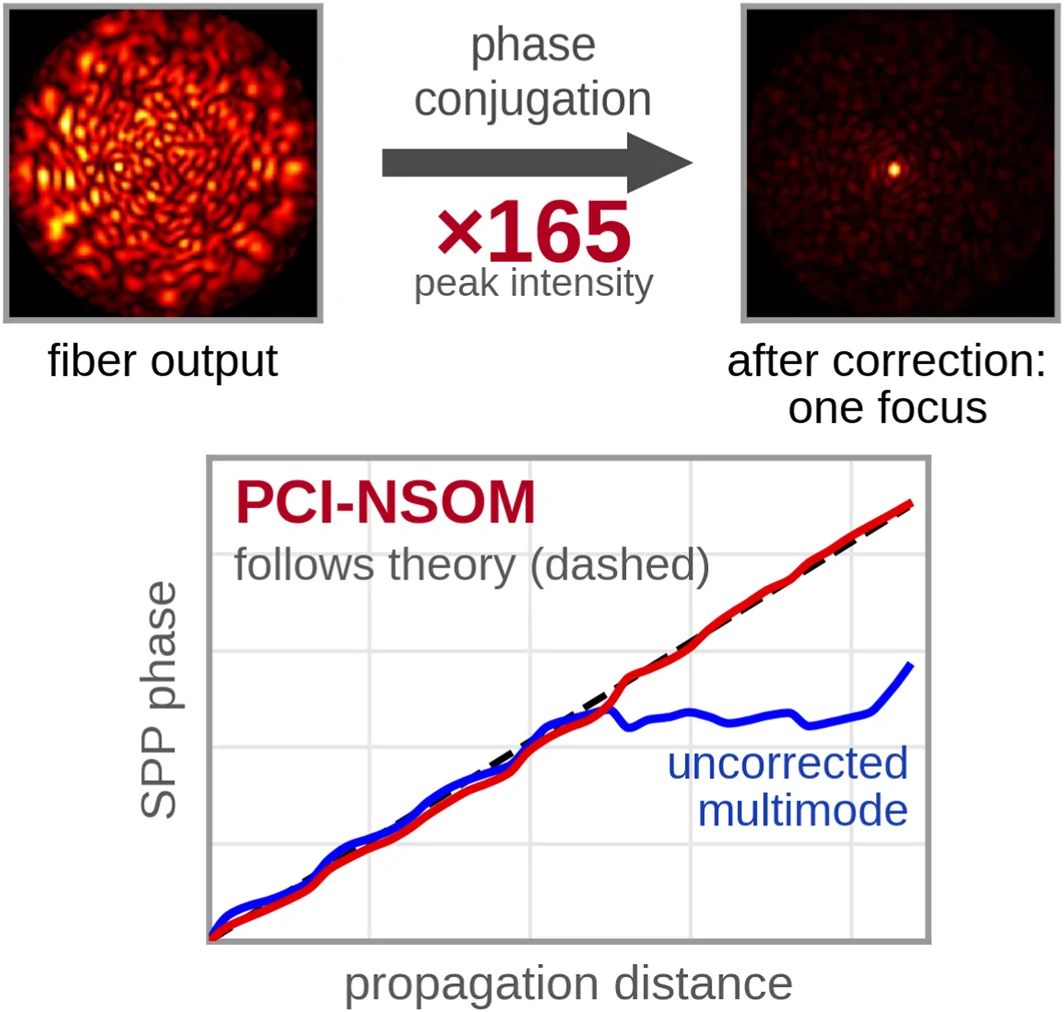

## 1 Introduction

Optical microscopy is an indispensable tool across the physical and life sciences, yet its spatial resolution is fundamentally limited by diffraction to approximately half the wavelength of light. Far‐field super‐resolution techniques have pushed biological imaging to the nanometer scale [1, 2, 3, 4, 5], but these methods rely on fluorescent labeling and specific photophysical contrast mechanisms, limiting their applicability to general nanophotonic characterization.

Near‐field scanning optical microscopy (NSOM) offers a different route to nanoscale optical imaging [6, 7, 8, 9]. By positioning a sub‐wavelength probe in the immediate vicinity of a sample surface, NSOM captures evanescent field components that encode sub‐wavelength spatial information, achieving a resolution determined by the probe geometry rather than by the wavelength of light. The probe is either a sub‐wavelength aperture at the end of a tapered fiber, through which the local field is collected (aperture‐type NSOM) [10], or a sharp metallic tip that scatters the local field into the far field (scattering‐type NSOM) [11]. Since its early development [6, 7], NSOM has evolved into a versatile platform with applications spanning single‐molecule detection in biological environments [12], plasmonic near‐field nano‐imaging [13], supported by steady advances in probe design, force feedback, and integration with other imaging modalities [14]. In particular, NSOM has become a central tool for studying surface plasmon polaritons (SPPs) [15, 16], enabling direct visualization of SPP propagation, interference, and scattering at the nanoscale [17, 18, 19] as well as ultrafast imaging of localized plasmon dynamics by plasmonic nanofocusing [20].

In standard intensity‐based NSOM, only the modulus of the near‐field signal is recorded, whereas many nanophotonic phenomena, among them wave propagation, interference, vortex formation, and mode coupling, require both the amplitude and the phase of the optical near field. Interferometric NSOM addresses this need by incorporating the near‐field probe into an interferometric detection scheme. It has been implemented in both modalities of NSOM, and the two differ in what they measure. In scattering‐type NSOM, amplitude‐ and phase‐resolved detection of tip‐scattered light was first demonstrated by combining heterodyne interferometry with higher‐harmonic demodulation of the tip oscillation [21], later refined into pseudo‐heterodyne detection [22] and synthetic optical holography [23]. Scattering‐type NSOM provides complex‐valued near‐field signals from the visible to the terahertz range with a spatial resolution of 10–100 nm, set by the tip apex radius [11]. Examples include the phase‐resolved mapping of plasmonic antenna near fields [24] and of propagating SPPs on gold platelets [25], and the amplitude imaging of plasmon standing waves in carbon nanotubes [26]. In this modality, the tip typically both launches SPPs and scatters them into the detector, so the recorded signal is generally the interference between the near‐field response of the tip and the polaritons launched by the tip or by the sample.

In aperture‐type NSOM, the field collected through the aperture is brought to interference with a reference as in the early pseudoheterodyne [27] and heterodyne [28] schemes and in phase‐shifting holography [29]. Since the aperture collects the local field without appreciably launching SPPs, the measured phase is to good approximation the propagating phase of the field on the sample. This has enabled the direct mapping of SPP interference and propagation [18], the simultaneous detection of electric and magnetic field components [30], the full vectorial mapping of nanophotonic light fields [31], and quantitative two‐dimensional near‐field phase imaging [32], establishing phase‐sensitive near‐field microscopy as an indispensable tool in nanophotonics [10]. The price is a coarser spatial resolution, set by the aperture rather than by a tip apex, and a collected signal limited by the throughput of the aperture–fiber channel.

In aperture‐type interferometric NSOM, the central limitation is the trade‐off between signal strength and phase measurement fidelity. Conventional systems employ single‐mode fiber (SMF) probes, which support a single spatial mode and thereby preserve the well‐defined phase relationship required for interferometric detection [27, 28, 32]. However, the small mode‐field diameter of the SMF severely limits the optical throughput, particularly in combination with the sub‐wavelength aperture needed for high spatial resolution. The resulting low signal‐to‐noise ratio (SNR) constrains the accuracy of near‐field phase measurements, especially for weakly scattering or low‐emission samples. Multimode fiber (MMF) probes, with their larger core diameter and multitude of propagation modes, offer substantially higher optical throughput, but the differential phase velocities of the many spatial modes produce a randomized speckle pattern at the fiber output, destroying the phase fidelity necessary for interferometric detection.

Overcoming the modal mixing in MMF has been a central pursuit of the wavefront shaping community [33]. Early work demonstrated that coherent light can be focused through strongly scattering media by iterative optimization with a spatial light modulator (SLM) [34] or by transmission matrix measurement [35], and the same approach was extended to the control of surface plasmons on a disordered metal film [36]. These principles were rapidly adopted for MMF: the transmission of a fiber was characterized and used to shape the light at its output [37], alongside holographic micromanipulation [38], scanner‐free wide‐field endoscopic imaging [39], diffraction‐limited focusing by digital phase conjugation [40], and high‐resolution lensless endoscopy [41, 42]. Subsequently, the modal transformation of a fiber was predicted from its geometry, including bends [43], and the approach was carried to in vivo deep‐brain imaging [44]. More recently, wavefront shaping concepts have been brought into the near‐field regime, where transmission matrix microscopy was used to map high‐order eigenmodes of sub‐wavelength nanostructures [45].

In this work, we present phase‐conjugation interferometric NSOM (PCI‐NSOM), a method that resolves the trade‐off between signal strength and phase fidelity in aperture‐type near‐field optical microscopy. An SLM displays the phase conjugate of the output field of the MMF probe, converting the scrambled speckle pattern into a planar wavefront that focuses to a single point in Fourier space. The correction requires a single hologram of the fiber output rather than a measurement of the transmission matrix of the fiber, and this output is invariant over the 20 × 20 μm^2^ range of the probe scan, so a single calibration serves an entire image. We demonstrate PCI‐NSOM by imaging SPPs on a gold grating nanostructure, obtaining both amplitude and phase maps with an approximately 66‐fold intensity enhancement over uncorrected MMF integral detection and approximately 133‐fold over a numerically projected SMF probe. The enhanced SNR enables reliable phase recovery in weak‐signal regions where uncorrected detection fails, establishing PCI‐NSOM as a practical tool for high‐sensitivity phase‐resolved nanoscale optical characterization.

## 2 Results

### 2.1 Working Principle of Phase‐Conjugation Interferometric NSOM

Aperture‐type interferometric NSOM has employed SMF for quantitative phase measurements, owing to its well‐defined spatial mode. However, the extremely low optical transmission efficiency inherent to sub‐wavelength apertures imposes a fundamental constraint on the collected signal, leading to a fundamental trade‐off between spatial resolution and SNR. In particular, when the aperture size is reduced to maximize resolution, the collected optical intensity decreases drastically, rendering reliable phase recovery impractical.

To overcome this physical limitation, we employ an NSOM probe based on an MMF. The MMF supports a large number of propagation modes, providing much higher optical collection efficiency compared to SMF. However, the interference among modes with different phase delays produces a severely distorted wavefront in the form of a speckle pattern at the fiber output (Figure 1a). As a result, the precision and reliability of direct phase recovery are compromised. In this work, we correct these phase distortions using an SLM to apply the phase conjugate of the MMF output field, thereby significantly enhancing the precision and sensitivity of phase detection. The key insight is that although the MMF introduces complex modal phase distortions, these distortions are deterministic for a given fiber configuration and can therefore be precharacterized and compensated. By converting the scrambled speckle into a planar wavefront, the signal is concentrated into a single focal spot in Fourier space, simultaneously restoring accurate phase recovery and dramatically improving the SNR.

**FIGURE 1:**
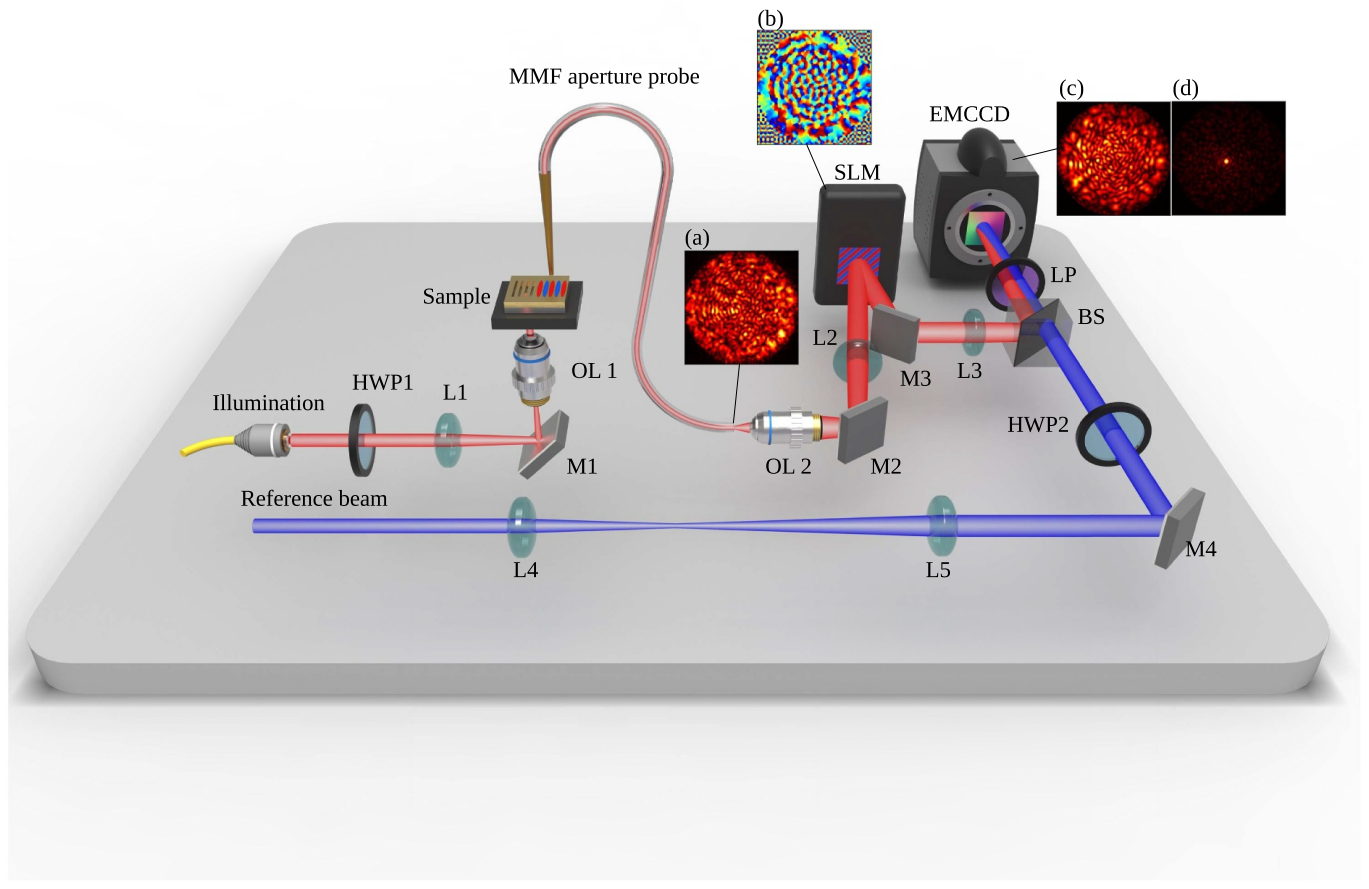
Interferometric near‐field scanning optical microscopy (NSOM) with wavefront correction. Schematic of the interferometric NSOM using a multimode fiber (MMF) aperture probe with wavefront correction. An illumination beam (colored in red) illuminates the sample from the bottom and the MMF aperture probe captures near‐field waves on the top. A reference beam (colored in blue) is introduced to the camera. OL 1, 2: objective lenses, SLM: spatial light modulator, EMCCD: camera, L1–5: lenses, M1–4: mirrors, BS: beam splitter, HWP1, 2: half‐wave plates, and LP: linear polarizer. (a) Typical speckle field at the MMF output, (b) correction phase map displayed on the SLM, (c) image before wavefront correction, and (d) image after the correction.

### 2.2 Experimental Setup of Phase‐Conjugation Interferometric NSOM

The experimental setup of PCI‐NSOM is shown in Figure 1. The complete optical layout and the detailed measurement parameters are given in Supporting Information S1: Figures S1 and S2 and Notes S1 and S2. We integrate an interferometric detection system into a commercial NSOM platform (Nanonics MV2000). The output beam of a laser diode (*λ* = 639 nm, Thorlabs LP637‐SF70) is split into a sample illumination beam and a reference beam. The illumination beam is focused onto the sample through an objective lens from below for transmission measurements or from above for reflection measurements, and the near‐field signal is collected by the MMF probe tip positioned in the near‐field region above the sample surface. The probe is a cantilevered fiber probe mounted on a tuning‐fork‐based normal‐force (intermittent‐contact) feedback system for distance regulation, maintaining an average tip–sample separation of approximately 10 nm during scanning. The typical scan step size is 125 nm, and the scan area spans several micrometers. The probe aperture diameter is 150 nm for the near‐field measurements reported here, and 250 nm for the throughput comparison of Supporting Information S1: Note S4. The multimode fiber used in our system (Corning ClearCurve OM2, core diameter 50 μm, and NA = 0.20 ± 0.015) has a *V*‐number of approximately 49 at 639 nm, supporting an estimated ∼600 guided modes, and a length of approximately 1 m. The scanning rate is 100 points per second.

The collected near‐field signal propagates through the MMF and exits at the distal end as a speckle field (Figure 1a). To measure and compensate for the phase distortion of this speckle field, we designed the detection system as follows. The MMF output beam is relayed to an SLM (Hamamatsu LCOS‐SLM X10468) positioned at a plane conjugate to the MMF output. The beam reflected from the SLM is then directed to an electron‐multiplying CCD (EMCCD) camera (iXon Ultra 897) placed at the corresponding Fourier plane, where the complex field—comprising both amplitude and phase—is measured via off‐axis holographic imaging. A reference beam is introduced to the camera for interferometric detection. Half‐wave plates (HWP 1, 2) are used to control the polarization states of the sample and reference beams, and a linear polarizer (LP) is placed in front of the camera to ensure polarization matching between the two beams.

The measurement procedure consists of two steps. In the first step, with no sample in place, a flat phase pattern is displayed on the SLM and the speckle field is recorded by the camera. The complex field at the MMF output plane is then obtained by Fourier transforming the measured k‐space data, and the conjugate of its phase (Figure 1b) is computed and displayed on the SLM. This phase compensation converts the speckle field (Figure 1c) into a focused spot at the center of k‐space (Figure 1d), thereby improving the precision of phase measurement and enhancing the SNR by concentrating the photons into a single point. In the second step, with the correction pattern applied, either the sample or the probe is raster‐scanned by a piezoelectric stage. At each scan position, the amplitude and phase are extracted from the holographic measurement at the camera from which the interferometric NSOM image is constructed.

### 2.3 Spatial Invariance of the Multimode Fiber Output

For the proposed method to work, the speckle field at the MMF output plane must remain invariant as the probe scans across the sample surface. Only then does the correction pattern obtained in the first step remain valid throughout the NSOM imaging session. This condition is readily satisfied because the scan range is small relative to the fiber length, and we have optimized the mechanical stability of the system.

To experimentally verify this speckle field stability, we performed a proof‐of‐concept experiment using a single‐slit sample (Figure 2a). The slit was illuminated from below, and the near‐field signal was collected by raster‐scanning the NSOM probe, recording the speckle field at the camera for each probe position. Figure 2b shows a conventional intensity‐based NSOM image obtained by integrating the total intensity of the field measured at the camera as a function of scan position. Figure 2c displays the complex fields of the MMF output extracted at two spatially separated positions (Position A and Position B) within the measurement area. The amplitude maps are nearly identical, and the phase maps also agree closely except for an overall phase offset. This global phase offset originates from path‐length fluctuations in the interferometric detection and varies negligibly within each line scan, allowing it to be removed in the image processing stage.

**FIGURE 2:**
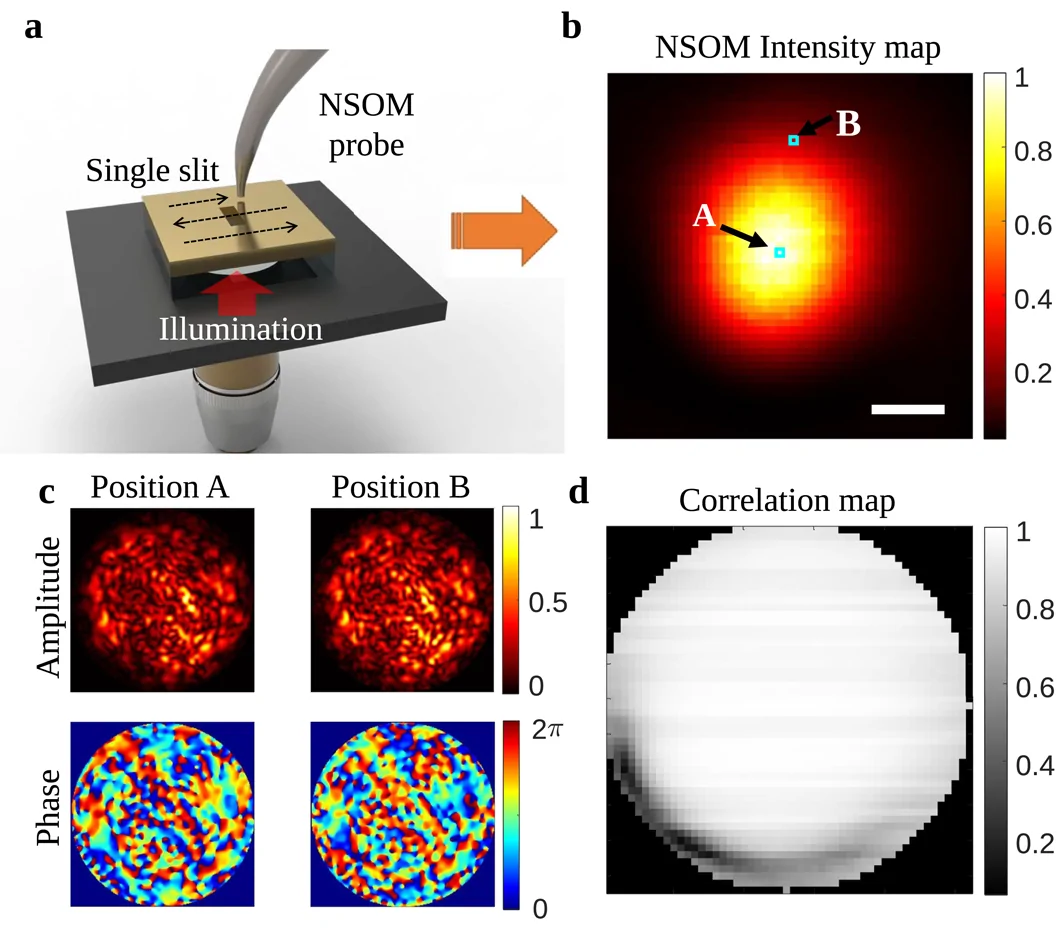
Spatial invariance of the MMF output during probe scanning. (a) NSOM scanning schematic. (b) Near‐field intensity map obtained by raster‐scanning the MMF probe across the sample surface. Color bar: normalized intensity. Scale bar: 200 nm. (c) Amplitude and phase distributions of the MMF output speckle field recorded at the camera for two spatially separated probe positions (Position A and Position B). Each map spans the numerical aperture of the fiber (NA = 0.20). Color bars: normalized amplitude (left) and phase in radians (right). (d) Normalized correlation of the MMF output fields across the entire scanning area in (b) with respect to the output field at the center position.

For a more quantitative analysis, we computed the two‐dimensional spatial correlation map of the speckle patterns across the entire scanning region (Figure 2d). The correlation exceeds 0.96 across the region in which the collected signal exceeds half its maximum, and it decreases toward the periphery of the scan in step with the local signal level rather than with the distance from the center, which identifies detection noise rather than a change of the fiber as its origin. To further examine the probe scan range over which the speckle remains invariant, we performed a dedicated 20 × 20 μm^2^ probe scan, in which the correlation of the fiber output field with that at the scan center stays above 0.98 over the whole field (Supporting Information S1: Figure S3). This confirms that the MMF probe constitutes a robust space‐invariant system whose internal mode coupling characteristics remain unchanged during probe scanning. Consequently, a single phase conjugation pattern computed at one particular position and applied to the SLM ensures that the wavefront correction remains valid over an area larger than a typical near‐field image, guaranteeing high‐sensitivity continuous near‐field scanning without the need for dynamic SLM updates.

Air currents and mechanical perturbation can slightly deform the approximately 1 m fiber that runs from the NSOM unit to the optical table, which changes the output speckle. We suppress this by gently fixing the fiber to the body of the NSOM and by enclosing the NSOM and the phase‐conjugation system together. Over a 10‐min record the correlation of the fiber output with the pattern captured at the start fluctuates between 0.78 and unity on the open optical bench, whereas it stays above 0.978 with the setup enclosed. Acquiring one near‐field image takes a few seconds, so a single calibration remains valid over many consecutive images. In practice, we renew it at the start of each measurement, which costs only a few seconds. The correction holds as long as the overall fiber conformation is unchanged, and rerouting or bending the fiber requires recalibration. The full analysis is given in Supporting Information S1: Note S3 and Figure S4.

### 2.4 Experimental Demonstration of Wavefront Correction and Signal Enhancement

Here, we describe the wavefront correction process introduced in the experimental setup section in greater detail. Figure 3a shows the optical layout for wavefront measurement and correction. The speckle field at the output plane of the MMF is relayed to the SLM plane via the objective lens and lens L1, and its Fourier transform is delivered to the camera via lens L2. The SLM thus operates in real space (r‐space), whereas the camera is positioned in spatial frequency space (k‐space). A reference beam is introduced to the camera to acquire the complex field map of the sample beam in k‐space.

**FIGURE 3:**
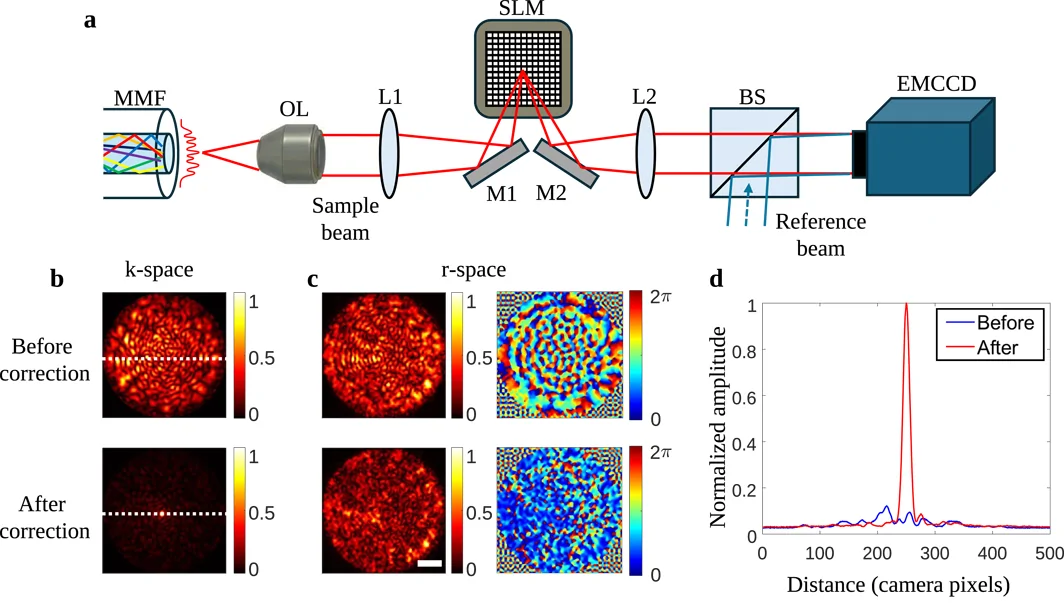
Experimental demonstration of active wavefront correction and interferometric signal enhancement. (a) Optical layout for the phase conjugation process. The SLM is located at a plane conjugate to the MMF output, and the EMCCD is located at its Fourier plane. The complex field is measured in k‐space by the EMCCD camera using off‐axis holography, and phase correction is applied in r‐space by the SLM. (b) Measured k‐space amplitude distributions before (top) and after (bottom) applying the phase‐conjugate correction. (c) Digitally reconstructed r‐space amplitude and phase distributions obtained via fast Fourier transform (FFT) of the k‐space complex field maps in panel (b). The severe, random spatial phase fluctuations (top, before) are successfully flattened into a uniform phase distribution (bottom, after). Scale bar: 10 μm. (d) Normalized amplitude profiles of the k‐space signals along the dashed lines in panel (b), demonstrating the significant signal enhancement by the wavefront correction.

The upper image in Figure 3b (before correction) shows a typical amplitude map of the MMF output measured in k‐space, exhibiting a speckle pattern characteristic of the MMF output. This k‐space map is then Fourier transformed to digitally reconstruct the complex field distribution in r‐space (Figure 3c, top). The amplitude map similarly displays a speckle pattern, and the phase map is distorted with values fluctuating between 0 and 2π. We applied the conjugate of this phase pattern to the SLM. As a result, the phase distribution in r‐space became spatially uniform (Figure 3c, bottom), and the measured field in k‐space was converted into a tightly focused spot on the camera (Figure 3b, bottom). In essence, the SLM converts the MMF output into a nearly planar wavefront that undergoes constructive interference and concentrates into a single focal point at the center of k‐space. Without phase correction, the light is broadly scattered across k‐space, degrading the accuracy of near‐field phase extraction and lowering the SNR.

We compared the amplitude profiles of the beam at the camera plane before and after correction (Figure 3d). The peak signal was enhanced by factors of 12.9 in amplitude and 165.9 in intensity. As demonstrated in the following section, this enhancement translates into a substantial improvement in SNR when imaging realistic samples with weak near‐field signals. For phase‐only correction over the ∼600 guided modes of our MMF (*V*‐number of approximately 49), the theoretical intensity enhancement is approximately 470 [34]. The measured value falls below this upper bound primarily because the optical power is not uniformly distributed among the guided modes together with residual imperfections in the wavefront measurement and SLM correction. In our experiments, this nonuniform mode excitation was the dominant factor limiting the achievable enhancement.

We note that this 12.9‐fold amplitude (165‐fold intensity) enhancement represents the photon‐concentration capacity of the wavefront correction for an input field that is broadly distributed across the MMF modes. For a real near‐field signal whose coupling into MMF modes is biased—for instance, weighted toward low‐order modes—the focal‐spot pixel already collects a non‐negligible fraction of the signal before correction, and the observed enhancement ratio is correspondingly smaller. The maximum gain is therefore set by the modal distribution of the input field, not by the wavefront correction itself.

### 2.5 Near‐Field Imaging of Surface Plasmon Polaritons

To demonstrate that our method enables high‐accuracy amplitude and phase detection on a real sample, we performed near‐field imaging of SPPs propagating on a gold grating (Figure 4). The SPPs were excited by the gold grating with a period of approximately 370 nm (Figure 4a). A 1 × 6 μm^2^ region to the right of the grating edge, where SPPs propagate, was scanned (red shaded rectangle) with a probe aperture diameter of 150 nm and a scan step of 125 nm, under an illumination power of 20 mW over a beam size of approximately 6 μm on the sample.

**FIGURE 4:**
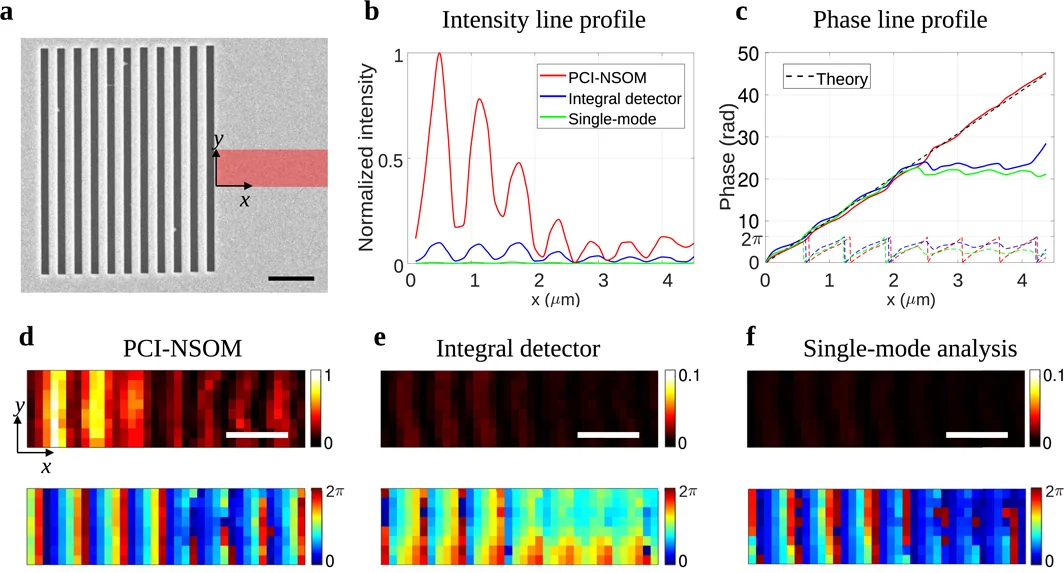
Near‐field imaging of surface plasmon polaritons on a gold grating. (a) SEM image of the Au grating (period ∼370 nm). The red rectangle marks the 1 × 6 μm^2^ scan region; *x* and *y* indicate axes corresponding to the SPP propagation and transverse directions, respectively. Scale bar: 1 μm. (b) Normalized near‐field intensity line profiles along the propagation direction (*x*). Red: PCI‐NSOM; blue: integral detector (uncorrected MMF); and green: numerically projected SMF probe. (c) Corresponding phase profiles (same color scheme as panel (b)). Solid: unwrapped phase; and dashed: wrapped phase. Black dashed line: theoretical SPP slope. Only PCI‐NSOM reproduces both the wrapped 0‐to‐2π ramp and the monotonic unwrapped phase consistent with the theoretical prediction. (d–f) Amplitude (top) and phase (bottom) maps obtained by (d) PCI‐NSOM, (e) uncorrected MMF integral detection, and (f) numerically projected SMF probe. Probe aperture: 150 nm; scan step: 125 nm. Scale bars: 1 μm. Top color bars: amplitude normalized to the maximum in panel (d). Bottom color bars: phase in radians.

The grating was fabricated by focused ion beam milling of a 100‐nm‐thick gold film deposited directly on glass. The conventional Ti/Cr adhesion layer was omitted to minimize ohmic damping and maximize the SPP propagation length, which is essential for evaluating phase fidelity in the heavily attenuated weak‐signal regime. The sub‐wavelength period (370 nm < λ = 639 nm) suppresses higher‐order propagating diffraction in free space and restricts SPP excitation to the high‐angle wavevector components of the illumination, thereby isolating the near‐field signal from the direct‐transmission background.

Before presenting the phase maps in Figure 4d, we briefly describe the postprocessing procedure used to remove low‐frequency global phase drift that accumulates during the raster scan. Within a single fast‐scan line (*x*‐axis), temporal phase fluctuations were negligible. However, low‐frequency environmental perturbations introduced global phase offsets between successive lines along the slow‐scan axis (*y*‐axis). To eliminate these temporal artifacts without distorting the genuine spatial phase progression of the SPPs, we exploited the one‐dimensional translational symmetry of the grating sample: the near‐field phase is expected to remain constant along any column parallel to the grating grooves. By selecting a single vertical column as a uniform phase reference, we calculated a zero‐order phase offset for each horizontal scan line to align the reference phase to a constant value. This line‐by‐line phase synchronization effectively removed the temporal drift, yielding the true two‐dimensional phase map of the propagating SPPs.

Figure 4d shows the amplitude and phase maps acquired after applying phase conjugation. At each scan position, the complex field values at the pixels constituting the corrected focal spot were coherently summed to yield the plotted values. The amplitude map reveals a signal that decays with propagation distance, consistent with the lossy nature of SPPs on gold. The phase map exhibits a periodic pattern in which the phase ramps from 0 to 2π and then resets, with a period matching the SPP wavelength, a clear signature of propagating SPPs.

For comparison, we obtained near‐field images using two conventional detection schemes. Figure 4e shows the amplitude and phase maps obtained using an integral detector—equivalent to a single‐element detector (e.g., a photomultiplier) placed directly behind the MMF probe, which coherently sums the entire fiber core output field shown in the upper panel of Figure 3c. Mathematically, this corresponds to reading the center pixel of the uncorrected k‐space map (upper panel of Figure 3b). Because the MMF's many guided modes carry different phase delays from modal dispersion, this coherent sum suffers from destructive interference, producing both a substantially reduced amplitude and a distorted phase that fails to reach the full 2π excursion expected for the propagating SPP. PCI‐NSOM avoids this problem by first applying the SLM phase conjugate to bring all modes into a common phase reference, so that the photons constructively interfere at the focal‐spot pixel.

Figure 4f shows the image that would be obtained with an SMF probe, computed by numerically projecting the MMF output field measured in r‐space (as in the top panel of Figure 3c) onto the fundamental Gaussian mode of a single‐mode fiber with a mode‐field diameter of 4.5 μm. We deliberately took the largest mode‐field diameter available among commercial visible‐band single‐mode fibers, so that the projection over‐estimates rather than under‐estimates the signal an SMF probe would collect. Therefore, the advantage quoted below is a lower bound. We emphasize that this is a computational projection of the same near‐field measurement, not an independent SMF acquisition. It serves as an approximate estimation of an SMF probe measurement. To verify the collection efficiency directly, we spliced a single‐mode fiber to the distal end of the same aperture probe and measured a difference of about 300 times between the two collection channels (Supporting Information S1: Note S4 and Figures S5–S7). Whereas the integral‐detection failure of Figure 4e originates from modal‐dispersion‐induced destructive interference, the SMF probe captures only a single fiber mode of the available near‐field signal. Its absolute amplitude is therefore intrinsically small, and the resulting low SNR leaves the phase corrupted by detector noise in weak‐signal regions, again failing to reach the full 2π excursion despite the absence of modal dispersion.

Figure 4b directly compares the near‐field intensity line profiles along the SPP propagation direction for the three detection schemes. Near the grating edge, the SPP intensity recovered by PCI‐NSOM (Figure 4d) is approximately 66 times higher than that obtained with the uncorrected integral detector (Figure 4e) and approximately 133 times higher than that obtained with the numerically projected SMF probe (Figure 4f). Fitting a damped sinusoid to the corrected line profile yields a spatial period of 612 ± 5 nm (fit standard error), in close agreement with the theoretical SPP wavelength *λ*_SPP_ ≈ 610 nm calculated from the dielectric function of gold (*ε*_Au_ ≈ −11.5 + 1.2i) [46]. The phase profile (Figure 4c) clearly demonstrates the advantage of phase conjugation. After correction, the wrapped phase exhibits a clean 0‐to‐2π ramp across the entire 6 μm scan range, and the corresponding unwrapped phase increases linearly with a slope that matches the theoretical SPP dispersion (dashed line). In contrast, both the integral detector and the SMF projection maintain a recognizable 0‐to‐2π wrapped ramp only within the first ∼2.5 μm of propagation. Beyond this distance, the SPP signal is overwhelmed either by modal‐dispersion‐induced destructive interference (integral detector) or by detector noise (SMF projection), and the wrapped phase loses its sawtooth periodicity. Consequently, when these phase profiles are unwrapped, the integral‐detector and SMF‐projection curves deviate from linearity beyond ∼2.5 μm, whereas only the PCI‐NSOM unwrapped phase remains linear throughout the full scan. This confirms that phase conjugation enhances the fidelity of not only the amplitude but also the phase measurement, particularly in the weak‐signal regions far from the SPP source.

## 3 Conclusion

We have demonstrated phase‐conjugation interferometric near‐field scanning optical microscopy (PCI‐NSOM), which overcomes the long‐standing trade‐off between signal strength and phase fidelity in interferometric near‐field measurements. A multimode fiber probe supplies the optical throughput, and phase conjugation on a spatial light modulator restores the phase fidelity that interferometric detection requires. A single phase‐conjugation pattern, measured once before imaging, cancels the modal dispersion and converts the scrambled multimode output into a planar wavefront. It remains valid throughout the scan because the MMF output is spatially invariant as we verified experimentally (Supporting Information S1: Note S3).

PCI‐NSOM achieves a focal‐spot intensity enhancement of up to ∼165‐fold. The gain realized on a given sample is set by the modal distribution of its near‐field signal at the fiber output. On the gold‐grating SPP sample, we obtained approximately 66‐ and 133‐fold intensity enhancements over uncorrected MMF integral detection and over a numerically projected SMF probe, respectively. This allowed the full 0‐to‐2π phase ramp of the propagating SPPs to be recovered across the entire 6 μm scan range, whereas the two conventional schemes preserve it only within the first ∼2.5 μm of propagation. The improvement costs no spatial resolution, which remains set by the probe aperture rather than by the fiber type.

The collected signal falls steeply with aperture diameter, so the smallest usable aperture, and hence the resolution, is set by the SNR. PCI‐NSOM relaxes this limit, because for a given aperture it collects more of the near field and delivers all of it as a single coherent mode. In an interferometric measurement, the phase error is about the inverse of the amplitude SNR. Against a strong reference, the amplitude SNR is the square root of the number of collected photoelectrons. The 150 nm aperture of Figure 4 delivered about 3 × 10^3^ counts per hologram, an amplitude SNR of about 60 and a phase error of about 0.02 rad. With the aperture transmission specified by the probe manufacturer Nanonics (about 10^−5^, 10^−4^, 10^−3^, and 10^−1^ at 50, 100, 200, and 500 nm), the amplitude SNR falls to about 30 at 100 nm and 10 at 50 nm for the same 10 ms dwell time. A 50 nm aperture therefore remains usable at the present scan speed, and a dwell time of about 0.4 s restores the SNR of the 150 nm measurement, so that a practical resolution of 50–100 nm should be attainable.

We also measured the throughput of the multimode channel against that of the same probe with a single‐mode fiber fusion‐spliced to its distal end (Supporting Information S1: Note S4 and Figures S5–S7). The measured ratio of about 300 matches approximately the ratio of the mode counts of the two fibers, which sets the upper limit of the advantage. The 133‐fold figure obtained on the SPP sample is smaller because a given near field populates only a subset of the MMF modes, and a field confined to fewer modes overlaps the single SMF mode more strongly. Therefore, single‐mode collection would leave about 10 counts per hologram under the conditions of Figure 4, and matching the SNR of the multimode channel would require roughly 300 times the illumination or the dwell time, beyond what the sample tolerates.

The scan area is bounded by the probe scan range over which the MMF output speckle remains invariant. The speckle pattern is unchanged over 20 × 20 μm^2^ (Supporting Information S1: Figure S3), so at least this area can be covered by a single calibration, and larger areas by intermittent recalibration at negligible time cost. The present imaging speed of 100 points per second is close to the maximum of 125 points per second of the NSOM platform, so it is set by the probe scanner rather than by the camera frame rate. The corrected signal is concentrated in a single spot, so the camera can be read out over a very small region of interest. However, with smaller apertures, the SNR becomes the limit.

The phase‐conjugation principle is independent of wavelength as long as the MMF probe supports multiple propagating modes. However, spectroscopic use is limited because the modal dispersion of the fiber is wavelength dependent, so a single correction holds only within a limited spectral band around the calibration wavelength. That band accommodates the narrow line of our laser diode but not a broadband source. Broadband or multi‐wavelength operation would require spectral multiplexing of corrections or a shorter fiber.

The calibration characterizes the fiber rather than the sample. The recorded fiber output field defines the mode that the probe captures, so PCI‐NSOM registers at each scan position the component of the near field that couples into this mode. Therefore, surface roughness does not invalidate the correction, and rough or strongly scattering samples merely reduce the near‐field signal coupled into the probe and hence the attainable SNR.

Looking forward, we anticipate that PCI‐NSOM will serve as a practical tool for the characterization of a wide range of nanophotonic systems, including plasmonic devices, metasurfaces, and photonic crystals, where simultaneous amplitude and phase mapping at the nanoscale is essential for understanding light–matter interactions.

## Author Contributions

**Juntaek Oh:** conceptualization, methodology, software, data curation, investigation, validation, formal analysis, visualization, writing – original draft, writing – review and editing. **Young‐Ho Jin:** methodology, investigation, resources, writing – review and editing, writing – original draft, visualization, validation. **Sungsam Kang:** formal analysis, writing – review and editing. **Myung‐Ki Kim:** resources, supervision, writing – review and editing. **Wonshik Choi:** supervision, funding acquisition, project administration, writing – original draft, writing – review and editing, conceptualization.

## Conflicts of Interest

The authors declare no conflicts of interest.

## Supporting information


Supporting Information S1

## Data Availability

The data supporting the findings of this study are available from the corresponding author upon reasonable request.
