# Supplementary material for: Interferometric Near‐Field Scanning Optical Microscopy via Phase Conjugation of a Multimode Fiber Probe
Source: Nanophotonics. 2026 Oct 16;15(20):e70333. doi: 10.1002/nap2.70333 (PMC13675657; doi:10.1002/nap2.70333)
Supplement: Supplementary file 1 [file NAP2-15-e70333-s001.pdf]

Supplementary Information

Interferometric near-field scanning optical microscopy via phase conjugation of a multimode fiber probe

Juntaek Oh[1,+], Young-Ho Jin[1,+], Sungsam Kang[1], Myung-Ki Kim[2], and Wonshik Choi[1,*]

[1]*Department of Physics, Korea University, Seoul 02841, Korea*

[2]*KU-KIST Graduate School of Converging Science and Technology, Korea University, Seoul 02841, Korea*

[+]*These authors contributed equally to this work.*

[*]*E-mail: wonshik@korea.ac.kr*

Supplementary Note S1: Optical setup

Figure 1 of the main text presents the schematic of the setup. Figure S1 shows the layout of the phase-conjugation interferometric NSOM as built.

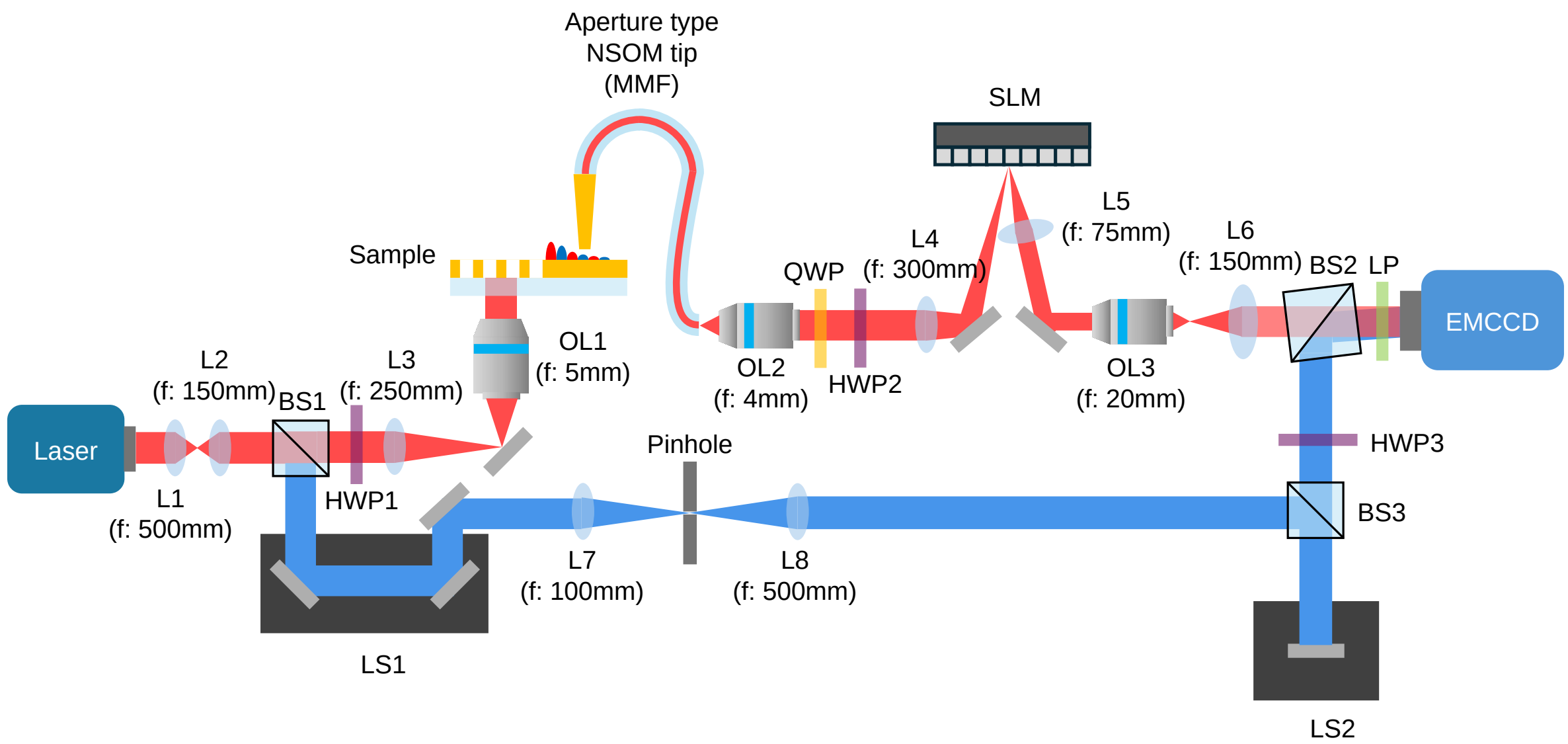

Figure S1. Optical layout of the phase-conjugation interferometric NSOM. Light from the laser diode (639 nm, Thorlabs LP637-SF70) is split by BS1 into a sample arm and a reference arm. The lenses L1, L2 and L3 expand and relay the illumination beam to the objective OL1. The sample arm illuminates the grating through the objective OL1, and the near field collected by the aperture of the multimode-fiber probe is captured by the $f = 4$ mm lens OL2 and imaged onto the spatial light modulator (SLM) by the $f = 300$ mm lens L4, a magnification of 75 that maps the 50 μm fiber core onto a 3.75 mm patch of the modulator. The modulated field is relayed by the $f = 75$ mm, $f = 20$ mm and $f = 150$ mm lenses (L5, OL3, L6) onto the EMCCD, where it is recombined at BS2 with the reference beam at an angle for off-axis holography. The half-wave plate HWP1 sets the illumination polarization to the sample. HWP2 and the quarter-wave plate QWP align the polarization of the sample beam emerging from the MMF with that of the SLM, and HWP3 aligns the reference beam with it. The linear polarizer LP passes only the component matched to the SLM polarization. L7, a pinhole and L8 spatially filter and expand the reference beam, and the beam splitters BS1 and BS3 feed the reference beam into the linear translation stages LS1 and LS2, which match its path length to that of the sample beam. The sample beam is drawn in red and the reference beam in blue only to distinguish them. Both are at the same wavelength.

Source, probe and fiber. The source is a single-mode fiber-pigtailed laser diode at $\lambda =$ 639 nm (Thorlabs LP637-SF70), split by a beam splitter into a sample arm and a reference arm. For the SPP measurement of Fig. 4 of the main text the sample arm illuminates the gold grating with 20 mW over a beam size of approximately 6 µm. The near field is collected through the sub-wavelength aperture (150 nm, while the throughput comparison of Note S4 uses a separate 250 nm probe) of a cantilevered, metal-coated tapered probe mounted on the tuning-fork normal-force feedback of a Nanonics MV2000 platform, which holds the average tip–sample distance at approximately 10 nm. The probe fiber is a Corning ClearCurve OM2 graded-index multimode fiber (50 µm core, NA 0.20 ± 0.015), which at 639 nm has $V \approx 49$ and supports approximately 600 guided modes including polarization. The collected signal is relayed from the NSOM scanning unit to the phase-conjugation system on the optical table, which makes the fiber approximately 1 m long.

Relay from the fiber to the camera. The fiber output is captured by the $f = 4$ mm lens, and the $f = 300$ mm lens images the fiber facet onto the spatial light modulator (Hamamatsu LCOS-SLM X10468, 792×600 pixels of 20 µm pitch) at a magnification of 75, so that the 50 µm core covers 3.75 mm, or 188 pixels, of the modulator. The $f = 75$ mm and $f = 20$ mm lenses relay the modulator plane to the front focal plane of the $f = 150$ mm lens at an overall magnification of $(300/4) \times (20/75) = 20$ from the fiber facet. There the core image is 1.0 mm across and the fiber numerical aperture is reduced to $\mathrm{NA}' = 0.20/20 = 0.01$. The camera sits at the back focal plane of the $f = 150$ mm lens, the Fourier plane of the fiber facet, where the numerical-aperture boundary falls at a radius of 150 mm $\times$ 0.01 = 1.5 mm, that is 94 pixels at the 16 µm pixel pitch of the EMCCD (Andor iXon Ultra 897, 512×512 pixels).

Supplementary Note S2: Off-axis holography and phase-conjugate correction

Recording the complex field. The reference arm is path-length matched to the sample arm to within the coherence length of the source (about 200 µm) and recombined with the signal at the camera at a small angle, which imposes a carrier fringe on the frame. The calibration hologram is acquired with an exposure time of 12 ms. The processing chain is shown in Fig. S2. A 200×200 pixel region of the hologram (Fig. S2a) containing the fiber numerical aperture (Note S1) is Fourier transformed (Fig. S2b). The autocorrelation term occupies a disc of 32 pixel radius at the origin and the two conjugate sidebands lie 104 pixels away along the diagonal. The first-order sideband is cropped with a circular mask of 16 pixel radius (Fig. S2c). This radius is set by the fiber: one pixel of this Fourier transform corresponds to 1.5 µm at the fiber facet, so the 50 µm core has a radius of 16.7 pixels. Demodulating the masked sideband to baseband gives the complex field at the camera plane (Fig. S2d), and its Fourier transform gives the field at the fiber facet (Fig. S2e). The phase of this field, resampled onto the modulator pitch, is the pattern whose conjugate is displayed on the spatial light modulator (Fig. S2f).

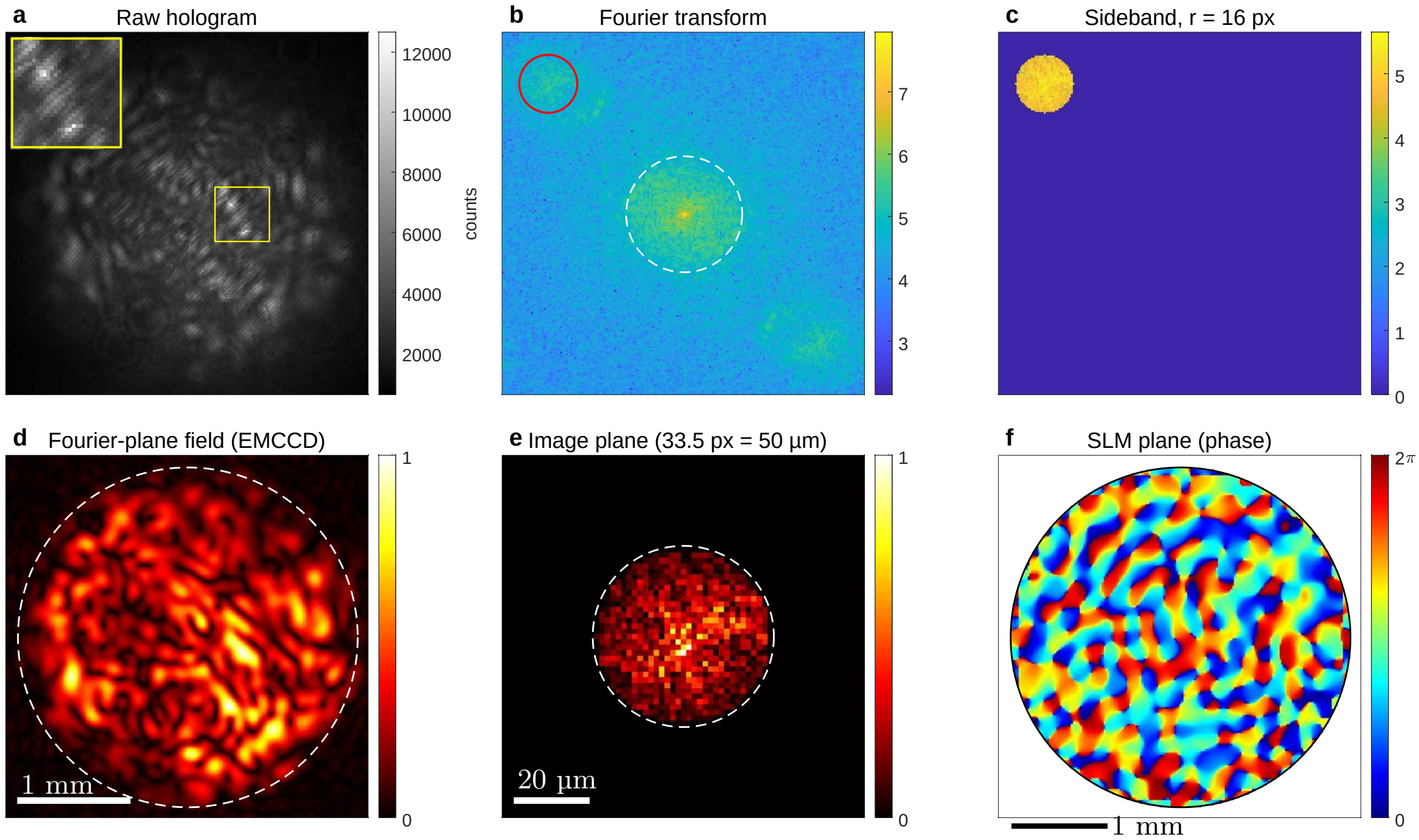

Figure S2. Retrieval of the fiber output field from an off-axis hologram. (a) Raw hologram recorded on the EMCCD at the Fourier plane of the fiber facet. The inset magnifies the boxed region four-fold to show the carrier fringes. (b) Fourier transform of a 200×200 pixel region (logarithmic scale). The autocorrelation term sits at the origin (white dashed circle) and the two sidebands 104 pixels away along the diagonal. The red circle is the 16 pixel mask applied to the first-order term. (c) Isolated sideband. (d) Amplitude of the field recovered at the camera plane. The dashed circle marks the fiber numerical aperture (NA = 0.20, 188 pixels in diameter). (e) Amplitude at the fiber facet, obtained by Fourier transforming (d). The 50 µm core spans 33.5 pixels. (f) Phase at the SLM plane, resampled onto the 20 µm modulator pitch, where the core spans 188 pixels within the 200×200 pixel correction region. Scale bars: 1 mm in (d), 20 µm in (e), 1 mm in (f).

Computing and applying the phase conjugate. With a flat pattern on the modulator and no sample in place, one hologram of the fiber output is recorded and the field at the fiber facet is retrieved as above. Its phase, resampled onto the modulator pitch (Fig. S2f), is negated to give the phase map to be displayed, which is converted into gray levels through the pre-calibrated phase response of the 256 gray levels of the modulator and uploaded. Since the core covers 188 pixels of the modulator (Note S1), the correction is written into a 200×200 pixel region containing this area. The computation takes less than one second, and the whole calibration, from hologram acquisition through data transfer to upload, is completed within a few seconds. It remains valid over many consecutive images under the stability conditions quantified in Note S3.

Supplementary Note S3: Stability of the multimode-fiber speckle pattern

A fixed correction pattern remains valid only as long as the fiber output field stays correlated with the field from which it was calibrated. Transmission through a multimode fiber depends on the conformation of the fiber, and the probe fiber is approximately 1 m long (Note S1). Two perturbations therefore have to be considered: the motion of the fiber end that accompanies the scanning of the tip, and the drift of the fiber between the NSOM unit and the optical table.

Tip scanning. Fig. 2 of the main text quantifies the first. The probe was raster-scanned over 1×1 µm^2 across the single-slit sample, which is illuminated from below and whose collected near-field distribution has a full width at half maximum of approximately 400 nm. The fiber output at every position was correlated with the output at the centre. Over the region in which the collected signal exceeds half its maximum the correlation stays above 0.96. Toward the periphery it decreases with the local signal level rather than with the distance from the centre, so the decrease is attributable to detection noise rather than to a change of the fiber. To determine the range directly, the illumination beam was widened and the probe was raster-scanned over 20×20 µm^2 (Fig. S3). Spatial frequencies outside the numerical aperture of the fiber, which carry only detection noise, were removed from each frame before the correlation was evaluated. The correlation of the fiber output field with that at the reference position in the center (marked by the cross in Fig. S3b) stays above 0.98 at every scan position, decreasing to 0.992 on average at the largest displacement of 14 µm (Fig. S3c). The output patterns recorded 11 µm from the reference position are almost indistinguishable from the reference pattern, with correlations of 0.993 to 0.998 (Fig. S3d). The fiber output field is therefore unchanged over at least the 20×20 µm^2 field measured here. The actual limit lies beyond it.

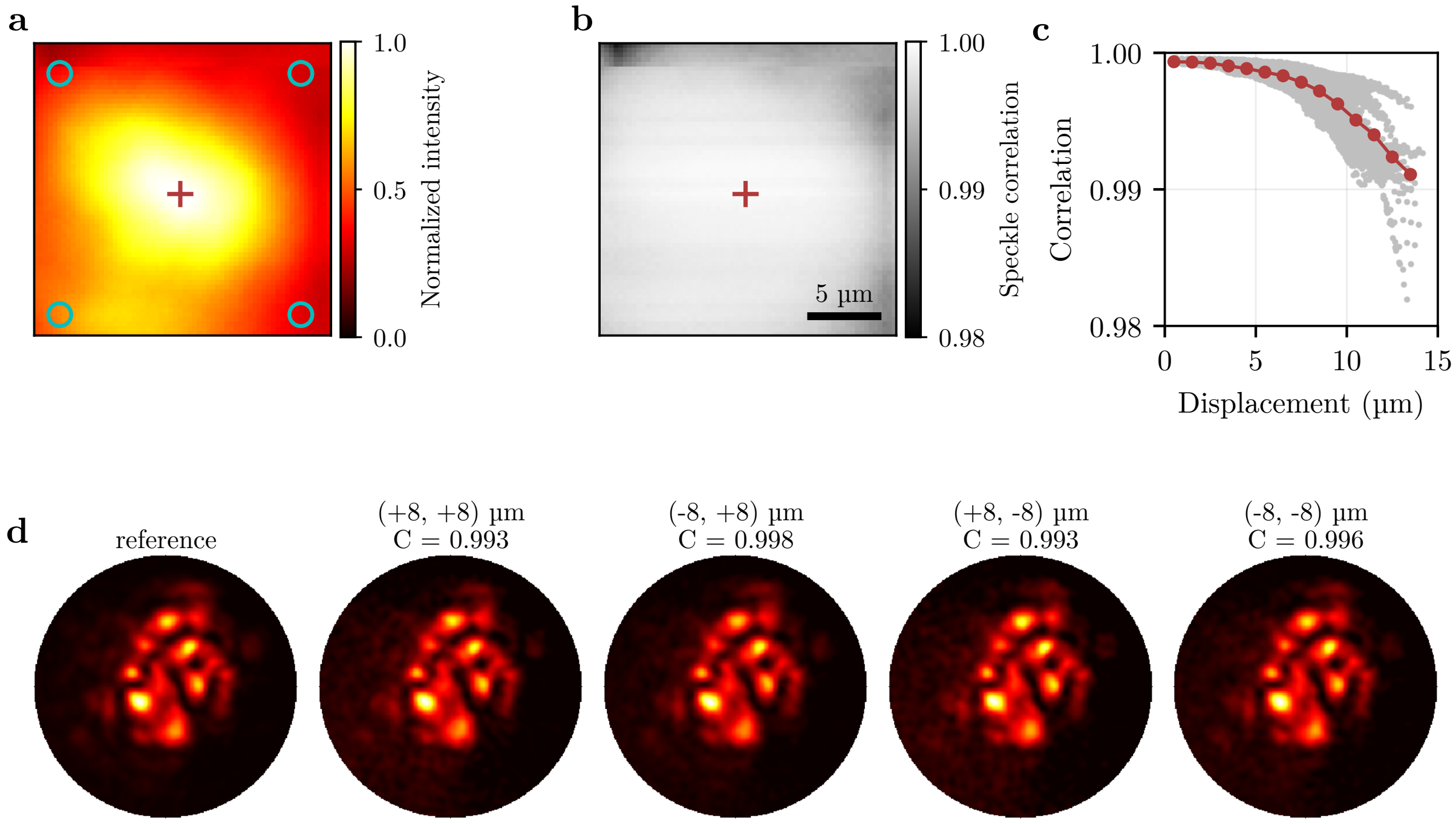

Figure S3. Invariance of the fiber output field under probe scanning. (a) Normalized near-field intensity collected while the probe was raster-scanned over 20×20 µm^2 across a widened illumination beam (8 mW, electron-multiplying gain 200). (b) Correlation of the fiber output field at each scan position with that at the reference position (cross), evaluated after each frame was low-pass filtered to the support of the fiber numerical aperture. (c) The same correlation against the displacement of the probe from the reference position (grey for all points and red for 1 µm bins). (d) Fiber output patterns at the reference position and at the four positions circled in (a), 11 µm from the reference, with their correlation values. Scale bar, 5 µm.

Drift. To suppress the second, the fiber is fixed to the body of the NSOM, which is enclosed together with the phase-conjugation system. To quantify the effect, the fiber output was recorded with the tip stationary for 10 minutes at one-second intervals and correlated with the pattern captured at the start of the record, on the open optical bench and with the enclosure in place (Fig. S4). On the open bench the correlation fluctuates, falling to 0.78 at its minimum and recovering to 0.98 at the end of the record, with a mean of 0.936. With the enclosure it stays above 0.978 throughout, with a mean of 0.986.

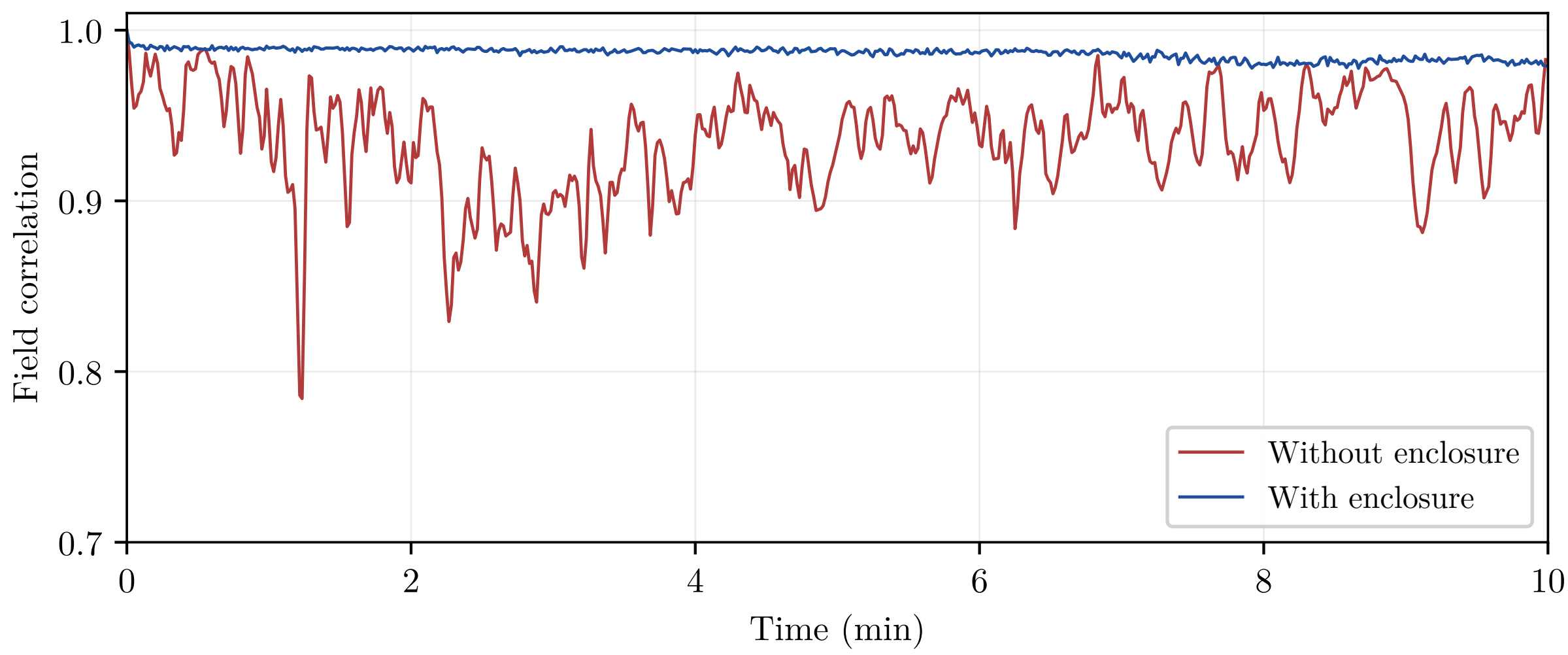

Figure S4. Stability of the multimode-fiber speckle pattern. Correlation of the fiber output field with the pattern recorded at $t = 0$, sampled every 1 s for 10 min on the open bench (red) and with the setup enclosed (blue). With the enclosure the correlation never falls below 0.978 over the record. The vertical axis is truncated at 0.70. Neither trace falls below that value.

A near-field image such as that of Fig. 4 takes about 4 s to acquire (384 points at a 10 ms dwell time). This is short compared with the 10-minute record, over which the enclosed correlation never falls below 0.978, so a single calibration remains valid over many consecutive images. The enclosed trace declines monotonically at about 0.09% per minute, which extrapolates to a correlation of 0.95 after roughly 45 minutes. Since one calibration takes only a few seconds (Note S2), it is renewed at the start of every measurement. Re-routing or re-bending the fiber invalidates the correction, and recalibration is then required.

Supplementary Note S4: Throughput of multimode versus single-mode collection

The advantage of multimode collection is quantified in the main text by projecting the measured multimode data onto a single-mode channel. This note reports the independent measurement against which that projection should be read.

Why the comparison is made on one probe. Ideally a single-mode and a multimode fiber probe would be compared directly, but the coupling efficiency of aperture probes often differs by an order of magnitude even at nominally the same aperture size. To remove this uncertainty, we compared one multimode aperture probe (aperture diameter 250 nm) with the same probe after a single-mode fiber (Thorlabs SM600, mode-field diameter 4.0 µm, NA 0.10–0.14) had been fusion-spliced to its distal end, so that the aperture, taper, metal coating and illumination are identical and only the collection channel differs (Fig. S5). The splice transmits 94% of the power in the single-mode-to-multimode direction.

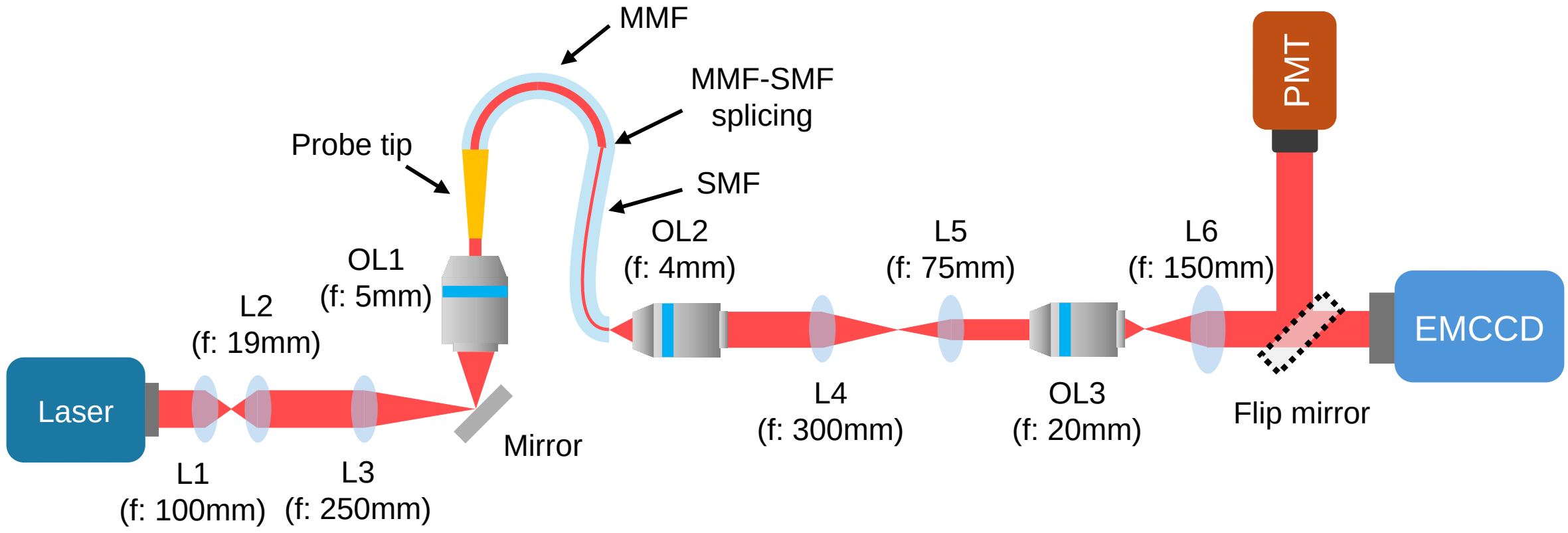

Figure S5. Configuration for the throughput comparison. The tapered aperture of the probe (aperture diameter 250 nm) is illuminated by a free-space beam through the objective lens OL1, and the light collected through the fiber is delivered by OL2 and the relay lenses to either a photon-counting module (PMT, Hamamatsu H8259-01) or an EMCCD (Andor iXon Ultra 897), selected by a flip mirror. The multimode fiber (MMF) of the aperture probe is measured directly and then again after a single-mode fiber (SMF, Thorlabs SM600) has been fusion-spliced to its distal end, so that only the collection channel differs. The aperture, taper, metal coating and illumination are identical in both cases. The core of the single-mode fiber is drawn thinner than that of the multimode fiber.

Photon-counting comparison. The total light delivered through the fiber was measured in each configuration with a photon-counting module (Hamamatsu H8259-01), by raster-scanning a 10×10 µm^2 region (Fig. S6). The background-subtracted integrated count rates are 13,650 kHz for multimode collection and 39.4 kHz for single-mode collection, a ratio of 346. Since both configurations share the same aperture, the ratio reflects the number of modes supported by the collection fiber. At 639 nm the multimode fiber supports about 600 guided modes including polarization (Note S1), whereas the SM600 supports two. The measured ratio thus matches approximately the value expected from the mode-count ratio and the 94% splice transmission. The 133-fold advantage predicted by the computational projection in the main text is lower because a given near field populates only a subset of the multimode-fiber modes, and a field confined to fewer modes overlaps the single SMF mode more strongly. The mode-count ratio therefore sets the approximate upper limit of the advantage.

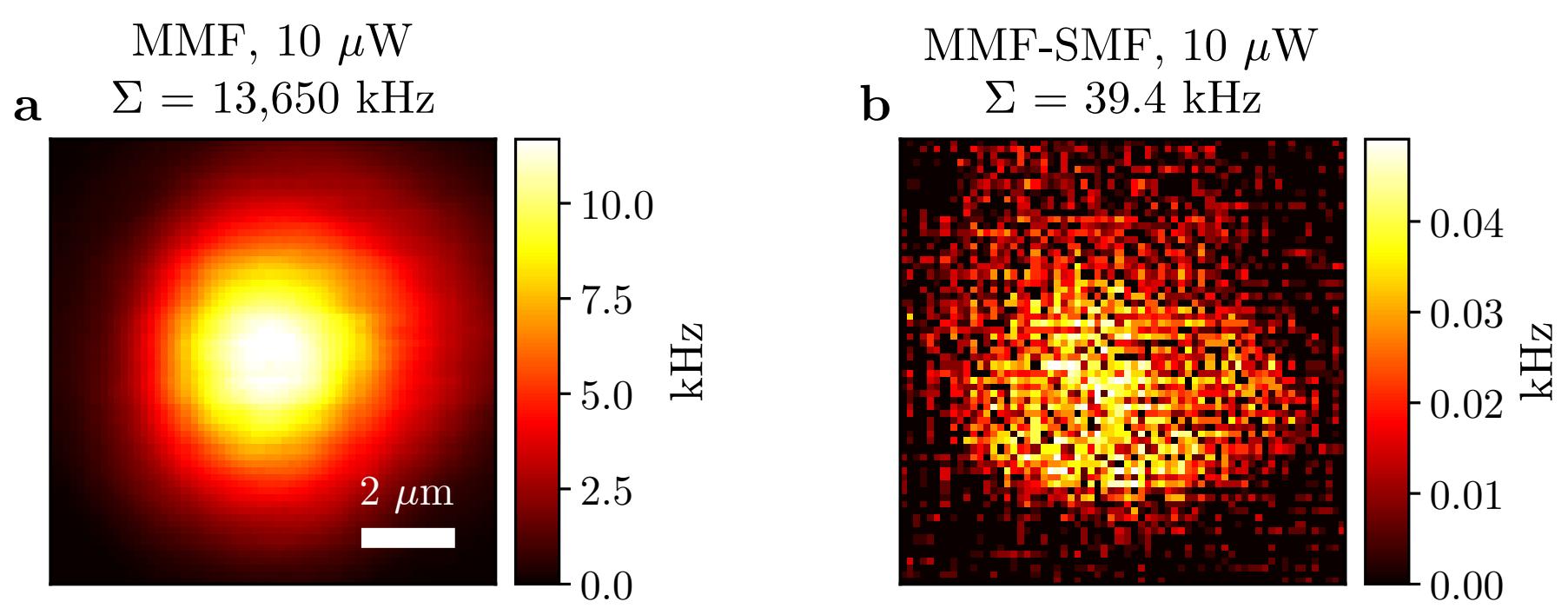

Figure S6. Throughput of multimode and single-mode collection, measured with a photon-counting detector. Background-subtracted count-rate maps recorded by raster-scanning the same 250-nm-aperture MMF probe over a $10 \times 10\,\mu m^2$ region under free-space illumination at $10\,\mu$W. Only the collection channel differs. (a) Direct MMF collection. (b) The same probe with a single-mode fiber spliced to its distal end. Σ in each title is the background-subtracted count rate integrated over the field: 13,650 kHz against 39.4 kHz, a ratio of 346. Colour scales are set independently for each panel. Dwell time 12 ms, counter integration 10 ms. Scale bar, $2\,\mu$m.

Camera-based comparison. The low coupling efficiency of single-mode collection is more detrimental to camera-based off-axis holography, where the signal must be resolved pixel by pixel rather than integrated on a single detector (Fig. S7). With multimode collection the output field is resolved on the EMCCD starting from 300 µW and fills the fiber numerical aperture. With the single-mode fiber spliced on, no output field is resolved at 300 µW or 1 mW, where the residual peaks of 64 and 71 counts are single noise pixels and no structure appears within the numerical aperture, although the photon-counting detector resolved this channel already at 10 µW (Fig. S6b).

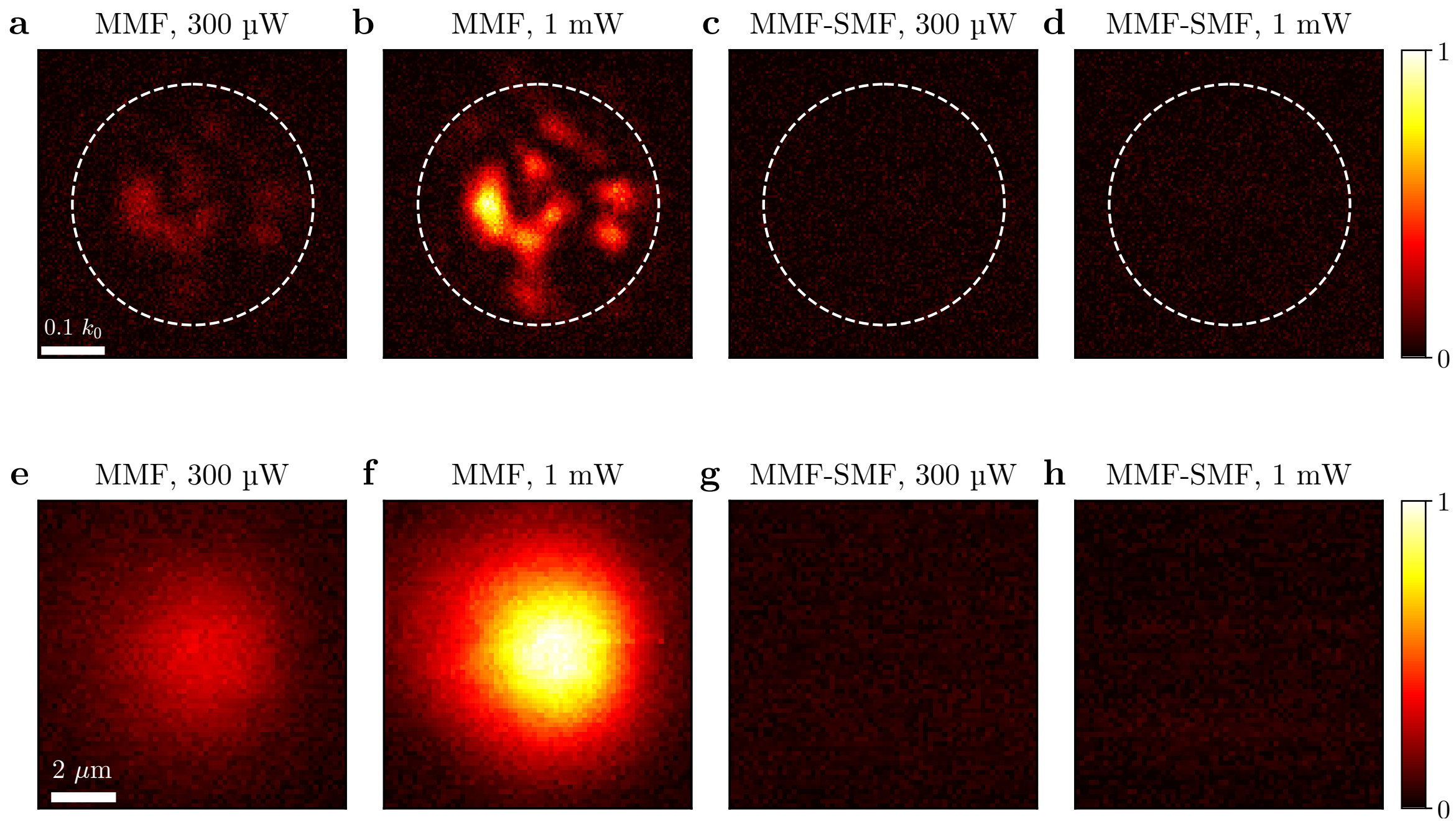

Figure S7. Camera-based detection with multimode and single-mode collection. (a–d) Fourier-plane images of the fiber output at the scan position of maximum signal, after background subtraction. The dashed circle marks the fiber numerical aperture (NA = 0.20). The camera noise is 14 counts per pixel. (e–h) The corresponding near-field maps over a 10 μm scan, obtained by integrating the signal within the fiber numerical aperture of each frame in (a–d). Columns show direct MMF collection at 300 μW and 1 mW, and the same probe with a single-mode fiber spliced to its distal end at the same powers. Each row shares one colour scale, set by its MMF, 1 mW panel. All panels were acquired without electron-multiplying gain and with an exposure time of 12 ms. Scale bars, 0.1 k_0 in (a) and 2 μm in (e).

Scaling to the imaging conditions. A measured difference in collection efficiency of about 300 is too large to be compensated by a modest increase in illumination. In the SPP measurement of Fig. 4 of the main text (150 nm aperture, 20 mW, 10 ms) the near field collected through the multimode probe amounts to about 3×10^3 counts per hologram within the fiber numerical aperture, about 6,000 times less than in the free-space geometry of Fig. S7b (1.1×10^6 counts at 1 mW and 12 ms) at equal illumination and exposure, reflecting the smaller aperture and the weaker near-field coupling. Single-mode collection would leave roughly 1/300 of this, about 10 counts per hologram. Since interferometric detection against a strong reference is shot-noise limited, the amplitude signal-to-noise ratio of the retrieved field is about the square root of the number of detected photoelectrons (about 1.2 electrons per count at the setting used): about 3 per scan point for single-mode collection against about 60 for the multimode channel. Matching the multimode quality would require roughly 300 times the illumination or the dwell time, about 6 W on the gold film or 3 s per point (about 20 minutes per image instead of 4 s), neither of which the sample or the measurement tolerates. The throughput

deficit of single-mode collection therefore translates directly into a loss of phase fidelity, which is the limitation that phase-conjugation interferometric NSOM resolves.
